# Decreased expression of the NKG2D ligand ULBP4 may be an indicator of poor prognosis in patients with nasopharyngeal carcinoma

**DOI:** 10.18632/oncotarget.14917

**Published:** 2017-01-31

**Authors:** Yuanji Xu, Lin Zhou, Jingfeng Zong, Yunbin Ye, Gang Chen, Yanping Chen, Xuehong Liao, Qiaojuan Guo, Sufang Qiu, Shaojun Lin, Honglin Chen, Jianji Pan

**Affiliations:** ^1^ Shengli Clinical Medical College of Fujian Medical University, Fuzhou, China; ^2^ Department of Radiation Oncology, Fujian Provincial Cancer Hospital, Fujian Medical University Cancer Hospital, Fuzhou, China; ^3^ Fujian Provincial Key Laboratory of Translational Cancer Medicine, Fuzhou, China; ^4^ Laboratory of Immuno-Oncology, Fujian Provincial Cancer Hospital, Fuzhou, China; ^5^ Department of Pathology, Fujian Provincial Cancer Hospital, Fuzhou, China; ^6^ State Key Laboratory for Emerging Infectious Diseases, Department of Microbiology and The Collaborative Innovation Center for Diagnosis and Treatment of Infectious Diseases, The University of Hong Kong, Hong Kong SAR, China

**Keywords:** U16-binding protein 4, nasopharyngeal carcinoma, prognosis

## Abstract

U16-binding protein 4 (ULBP4), a human ligand for natural killer group 2, member D (NKG2D) receptor on NK cells and subsets of T cells, is thought to activate anticancer immune responses. However, the expression pattern and prognostic effect of ULBP4 in nasopharyngeal carcinoma (NPC) has not been investigated. We first compared ULBP4 expression between archival 15 NPC tissues and 8 normal nasopharynx (NP) tissues using qPCR. Then ULBP4 expression among 111 NPC specimens was validated on immunohistochemical examination. In addition, the association of ULBP4 expression with clinical characteristics and survival outcomes was analyzed. Furthermore, the impact of ULBP4 expression in NPC cells on the cytotoxic activity of NK cells was investigated. Both mRNA and protein ULBP4 expressions of NPC tissues were significantly lower than those in normal NP tissues. However, no association of ULBP4 expression with clinical characteristics was observed. Patients with NPC having decreased expression of UBLP4 had significantly poorer overall survival (OS), progression-free survival (PFS), and distant metastasis-free survival (DMFS) than those with preserved levels of ULBP4. On multivariate analyses, low expression of ULBP4 was of borderline significance for OS, PFS, and DMFS (*P* = 0.060, 0.053, and 0.076, respectively). Further, LDH analysis demonstrated that the cytotoxic activitity of NK cells against C666-1 or 5-8F NPC cells with lenti-ULBP4 was considerably increased as compared to those with lenti-vector at various E/T ratios. Hence, restoration of ULBP4 expression may be a novel therapeutic strategy for treatment of NPC. However, further study is required to confirm these findings.

## INTRODUCTION

Nasopharyngeal carcinoma (NPC) is regarded as the most common head and neck cancer in Southeast Asia, especially in Southern China, with the incidence approaching 30 per 100,000 person-years [1]. The current consensus on treatment includes early treatment with radiotherapy alone, and concurrent chemotherapy combined with radiotherapy for locally advanced disease. Currently, the 5-year overall survival (OS) rate for early stage NPC can exceed 90% with use of intensity-modulated radiotherapy (IMRT) [2]. For locally advanced NPC, OS rates of 78.4%-80% have been reported with a combination of IMRT and chemotherapy [3, 4]. However, 20%~30% of patients with NPC develop treatment failure and distant metastasis [5, 6]. It is therefore necessary to explore novel therapeutic modalities to treat patients with NPC.

The role of immunotherapy in human cancers has attracted much attention. Natural killer (NK) cells and CD8+ T cells represent effector immune cells that detect and eliminate transformed tumor cells by innate and adaptive immunity, respectively [7]. The NK group 2, member D (NKG2D) serves as an activating immune receptor expressed on the surface of a vast majority of NK cells, CD8+ T cells, γδ T cells, and in certain subsets of CD4+ T cells [8], NKG2D could trigger cytotoxic activity of immune effector lymphocytes after binding to its distinct ligands, which consist of the MHC class I-related chain (MIC) family (MICA and MICB) and the UL16 binding protein or retinoic acid early transcript (ULBP/RAET) family (ULBP1-6) expressed on the surface of tumor cells. This is a key mechanism leading to the killing of infected and transformed tumor cells by NK and T cells [9, 10].

Stress-induced ULBP4 (RAET1E), a transmembrane molecule belonging to the ULBP family, has been described as a human ligand of NKG2D. It is known to be up-regulated on the surface of virus- or bacteria- infected cells [11, 12], and in several transformed tumor cells in ovarian, cervical, colon and liver cancers [12–14]. However, its expression in normal tissues is largely restricted, including skin and small intestine [11, 15]. ULBP4-NKG2D interaction can not only activate NK cell and NK-mediated cytotoxicity [11, 15, 16], but also serve to stimulate the cytotoxicity of CD8+ T cell [11], which suggests an influence of ULBP4 on both innate and adaptive immune responses. In addition, ULBP4 expression on Epstein-Barr virus (EBV)-infected peripheral blood cells was shown to enhance the cytotoxic effect of γδ T cell on tumor cells and EBV-infected B cells by binding to both TCRγδ and NKG2D [12]. This hints at the importance of ULBP4 in the elimination of EBV infection. The ubiqutious association between NPC and EBV infection is well-documented [17]. Thus, the expression level of ULBP4 on NPC cell surface may determine its antitumor efficacy, besides being of prognositic value in patients with NPC.

Currently, the tumor specific expression pattern of ULBP4 and its association with clinical outcomes has been reported for human colorectal, ovarian, breast, and cervical cancers [14, 18–20]. However, the expression status and clinical significance of ULBP4 in patients wtih NPC has not been investigated. In the present study, we retrospectively analyzed the expression pattern of ULBP4 in NPC specimens at the mRNA and protein levels prior to initiation of treatment, and assessed its prognostic relevance. In addition, the impact of ULBP4 expression in NPC cells on the cytotoxic activity of NK cells was also investigated *in vitro*.

## RESULTS

### Characteristics and treatment outcomes

Of the 111 NPC patients, 81 (73.0%) were males and 30 (27.0%) were females; the median age of patients was 48 years (range, 12 to 82 years). Histologically, 106 (95.5%) of the patients had WHO type III disease and 5 (4.5%) had WHO type II disease. According to the current 7^th^ UICC/AJCC staging system, 3 (2.7%), 35 (31.5%), 45 (40.5%), and 28 (25.2%) patients out of the total 111 NPC patients were categorized as stages I, II, III, and IV, respectively. Information on other variables including T classification, N classification, and chemotherapy is shown in Table 1. For the entire group, the 5-year OS, PFS, DMFS, and LRFS rates were 86.4%, 83.5%, 84.4%, and 95.2%, respectively. 6 patients were found to develop disease recurrence, of which 5 patients developed local relapse, 2 patients experienced regional relapse, and 1 patient had both local and regional relapse. In addition, 16 patients were found to develop distant metastasis. Finally, a total of 15 patients died: 9 of distant metastasis, 1 of local relapse, 1 of treatment complications, 3 of other medical conditions, and 1 of unknown reason.

**Table 1:** Associations between ULBP4 expression and clinical characteristics of patients with nasopharyngeal carcinoma

Characteristics	No. of patients	ULBP4 expression		χ^2^	*P*
		Low	High		
Total	111	79	32		
Gender				2.5	0.114
Male	81	61	20		
Female	30	18	12		
Age				0.087	0.768
≤48 years	60	42	18		
>48 years	51	37	14		
Histology				2.479	0.114
WHO II	5	2	3		
WHO III	106	77	29		
T classification				0.355	0.551
T1-2	50	37	13		
T3-4	61	42	19		
N classification				0.60	0.806
N0-1	85	60	25		
N2-3	26	19	7		
AJCC stage				0.178	0.673
I-II	38	28	10		
III-IVa+b	73	51	22		
Chemotherapy				0.237	0.626
No	13	10	3		
Yes	92	69	29		

Abbreviation: WHO: World Health Organization.

### ULBP4 was transcriptionally downregulated in NPC by qPCR

**Figure 1:**
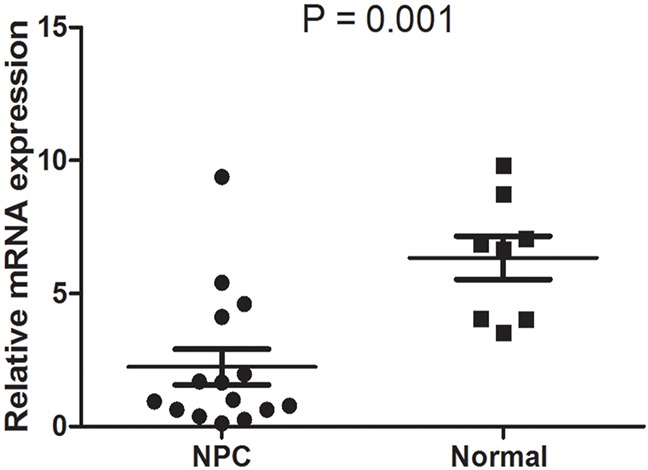
ULBP4 expression was decreased in NPC at mRNA level The mRNA expression of ULBP4 was significantly downregulated in 15 NPC tissue specimens as compared to that in 8 normal nasopharynx (NP) tissue specimens.

In order to determine the transcript expression levels of ULBP4, we analyzed the differences between 15 NPC and 8 normal NP fresh frozen tissue specimens by qPCR. It was found that the ULBP4 mRNA levels of NPC tissue specimens were significantly decreased as compared to that of normal NP tissue specimens (*P* = 0.001), as shown in Figure 1.

### ULBP4 expression was significantly downregulated in NPC at protein level on IHC

**Figure 2:**
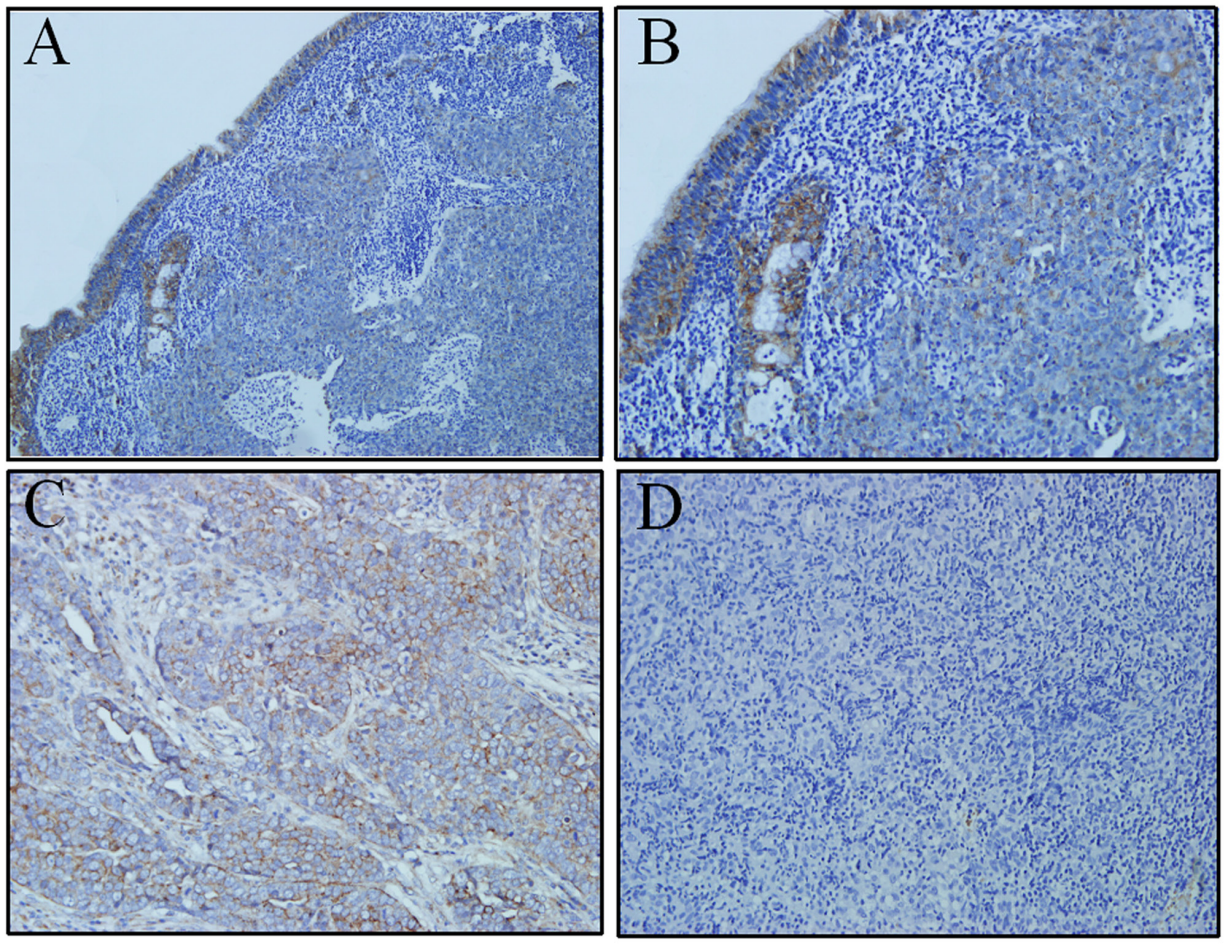
ULBP4 expression in adjacent normal epithelia and carcinoma cells as evaluated by immunohistochemistry analyses **A**. High ULBP4 expression was shown in adjacent normal naospharyngeal epithelia and low ULBP4 expression was shown in carcinoma cells (original magnificaiton ×100). **B**. High ULBP4 expression was shown in adjacent normal naospharyngeal epithelia and low ULBP4 expression was shown in carcinoma cells (original magnificaiton ×200). **C**. Positive control: ULBP4 expression was detected in human ovarian cancer tissue under the same conditions with anti-ULBP4 antibody (original magnificaiton ×200). **D**. Negtive control: NPC tissue was stained under the same conditions but anti-ULBP4 antibody was not used (original magnificaiton ×200).

On immunohistochemical analyses, 75 out of the 111 tumor samples had both tumor and normal epithelial tissues on the same slide. Therefore, ULBP4 expression levels were scored in both cancer tissues and the adjacent non-cancerous tissues. ULBP4 expression was lower in most cancer tissues when compared to the adjacent normal nasopharyngeal epithelial tissues (Figure 2). The IHC staining score 0-7 (≤ mean score of adjacent normal epithelial tissues) indicated low and the score 8-12 (> mean score of adjacent normal epithelial tissues) indicated high expression. Among these 75 tumor specimens, a high expression of ULBP4 in the adjacent normal nasopharyngeal epithelial tissues was found in 68% (51/75), whereas the expression in the corresponding cancer tissues was only in 26.7% (20/75) (χ^2^ = 25.7, P < 0.001) (Table 2). Overall, only 32 out of the 111 cancerous specimens had a high ULBP4 expression. These observations indicated the heterogeneity existing in the expression of ULBP4 of NPC tissues; however, the expression in NPC tissues was significantly lower in NPC tissues than that in the adjacent normal epithelial tissues.

**Table 2:** Comparison of ULBP4 expression between nasopharyngeal carcinoma and adjacent nasopharyngeal epithelia

Groups	ULBP4	ULBP4	χ^2^	*P*
	Low expression	High expression		
Carcinoma cells	55(73.3%)	20(26.7%)	25.7	<0.001
Adjacent epithelia	24(32%)	51(68%)		

### Association between expression of ULBP4 and clinical characteristics

To investigate whether the different expression of ULBP4 in NPC was significantly associated with clinical characteristics, all paraffin-embedded specimens were categorized based on different clinical characteristics. However, no significant association was found between ULBP4 expression and gender, age, pathologic type, T classification, N classification, clinical stage, and use of chemotherapy (Table 1).

### Association between expression of ULBP4 and clinical outcomes

**Figure 3:**
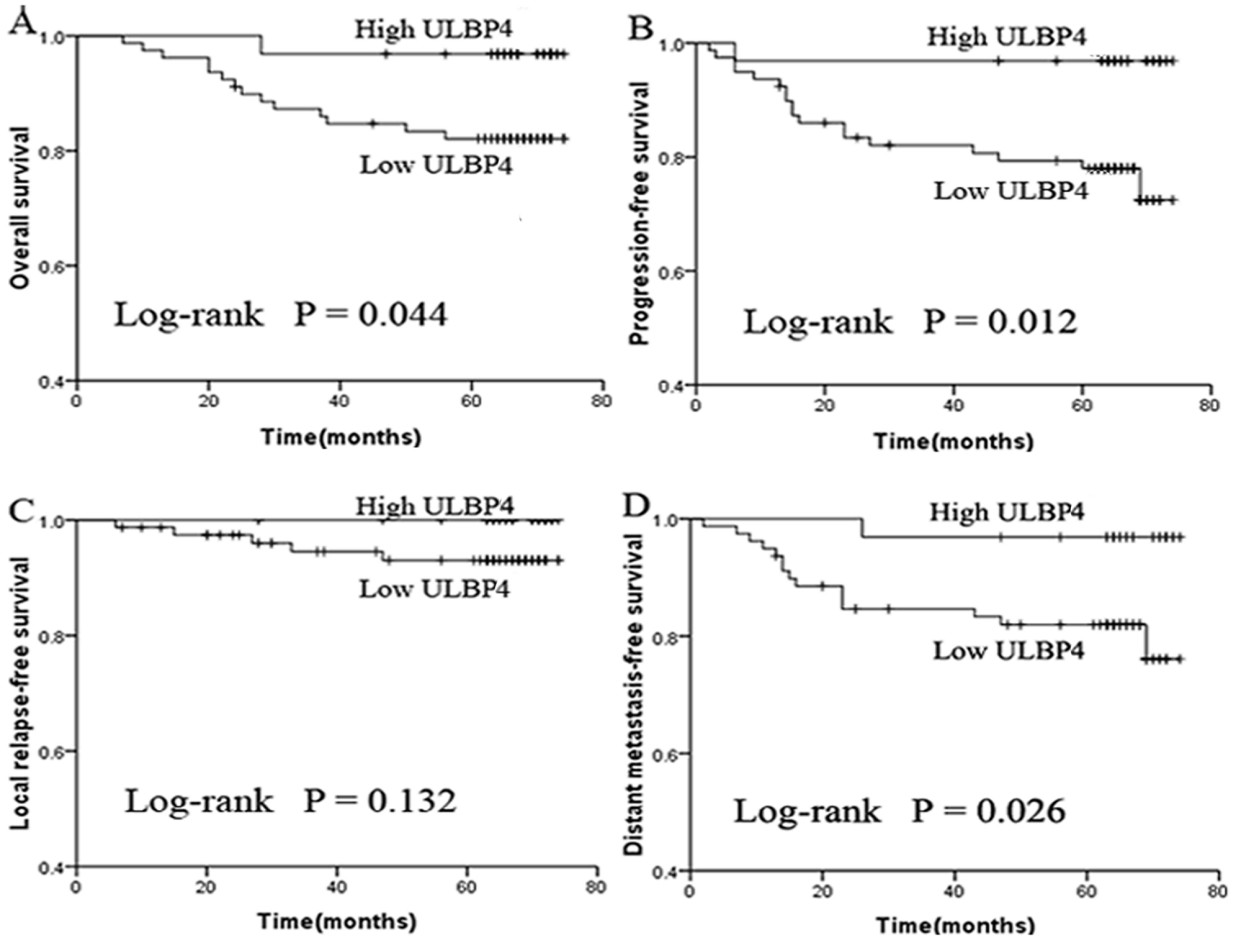
Low ULBP4 expression predicts inferior survival outcomes in patients with NPC The survival data was calculated and plotted by the Kaplan-Meier method. Patients were determined as low or high ULBP4 expression based on ULBP4 immunohistochemistry. *P < 0.05. **A**. Overall survival (OS). **B**. Progression-free survival (PFS). **C**. Local relapse-free survival (LRFS). **D**. Distant metastasis-free survival (DMFS).

To explore if the correlation between the differential expression of ULPB4 in NPC and the clinical outcomes, we made a comparison of survival outcomes, including 5-year OS, DFS, LRFS, and DMFS rates, between NPC patients with low and high ULBP4 expression, by univariate log-rank survival analysis. NPC patients with high ULBP4 expression had better 5-year OS, DFS, and DMFS rates compared to those with low ULBP4 expression (*P =* 0.044, 0.012, and 0.026, respectively) (Figure 3A, 3B, and 3D). However, ULBP4 expression in NPC had no statistically significant association with 5-year LRFS rates (Figure 3C). These results suggested that ULBP4 expression was negatively associated with tumor progression and distant metastasis. Additionally, other potential prognostic factors, such as gender, age, T classification, N classification, clinical stage, and use of chemotherapy, were also included to assess their correlation with clinical outcomes (Table 3). It was found that N classification correlated with both 5-year OS and DMFS rates, and females had a better 5-year DFS and DMFS rates than males.

**Table 3:** Potential prognostic factors by univariate log-rank analysis for patients with nasopharyngeal carcinoma

Factors	OS		PFS		LRFS		DMFS	
	Events	P*	Events	P*	Events	P*	Events	P*
ULBP4 expression		0.044*		0.012*		0.132		0.026*
Low (n=79)	14		18		5		15	
High (n=32)	1		1		0		1	
Gender		0.064		0.004*		0.155		0.010*
Male (n=81)	14		19		5		16	
Female (n=30)	1		0		0		0	
Age		0.905		0.458		0.492		0.651
≤48 (n=60)	8		9		2		8	
>48 (n=51)	7		10		3		8	
T classification		0.133		0.923		0.239		0.756
T1-2 (n=50)	4		9		1		8	
T3-4 (n=61)	11		10		4		8	
N classification		0.012*		0.070		0.986		0.018*
N0-1 (n=85)	8		12		4		9	
N2-3 (n=26)	7		7		1		7	
AJCC stage		0.066		0.316		0.089		0.319
I-II (n=38)	2		5		0		4	
III-IVa+b (n=73)	13		14		5		12	
Chemotherapy		0.789		0.362		0.423		0.490
No (n=13)	2		1		0		1	
Yes (n=92)	13		18		5		15	

Notes:*P < 0.05 indicates statistically significance among the variables. Events represent the numbers of failed cases for OS, PFS, LRFS, DMFS, respectively.

Abbreviations: OS: overall survival; PFS: progression-free survival; LRFS: local relapse-free survival; DMFS: distant metastasis-free survival.

### Low ULBP4 expression may be an independent predictor of poor prognosis

To further explore whether expression of ULBP4 has independent prognostic value in patients with NPC, multivariate Cox regression analysis was performed to avoid the interference among various prognostic factors. The following variables were adjusted in the Cox proportion hazards model by the backward elimination method: ULBP4 expression (low expression *vs*. high expression), age, sex (male *vs*. female), T classification (T1-2 *vs*. T3-4), N classification (N0-1 *vs*. N2-3), use of chemotherapy (no *vs*. yes). As shown in Table 4, N classification was found to affect both OS and DMFS (*P* =0.004 for OS and *P* =0.048 for DMFS). However, ULBP4 expression level was not significantly associatied with death or any type of treatment failure. Of note, low ULPB4 expression was found to have a borderline significance for death, disease failure, and distant failure (*P* = 0.060, 0.053, 0.076, respectively). These findings indicated that low ULBP4 expression may be an independent prognostic factor.

**Table 4:** Independent prognostic factors by multivariate cox-regression analysis for patients with nasopharyngeal carcinoma

Endpoint	Factors	P*	HR	95%CI for HR
Death	N classification	0.004*	4.832	1.674-13.946
	ULBP4 expression	0.060	0.142	0.018-1.087
	Age	0.022	1.045	1.006-1.086
Disease failure	N classification	0.098	2.241	0.854-5.883
	ULBP4 expression	0.053	0.132	0.017-1.026
Distant failure	N classification	0.048*	2.808	1.010-7.807
	ULBP4 expression	0.076	0.154	0.020-1.218

Notes: *P < 0.05 indicates statistically significance among the variables.

Abbreviations: HR: hazard ratio; CI: confidence interval.

### Impact of ULBP4 expression on the cytotoxicity activity of NK cells *in vitro*

**Figure 4:**
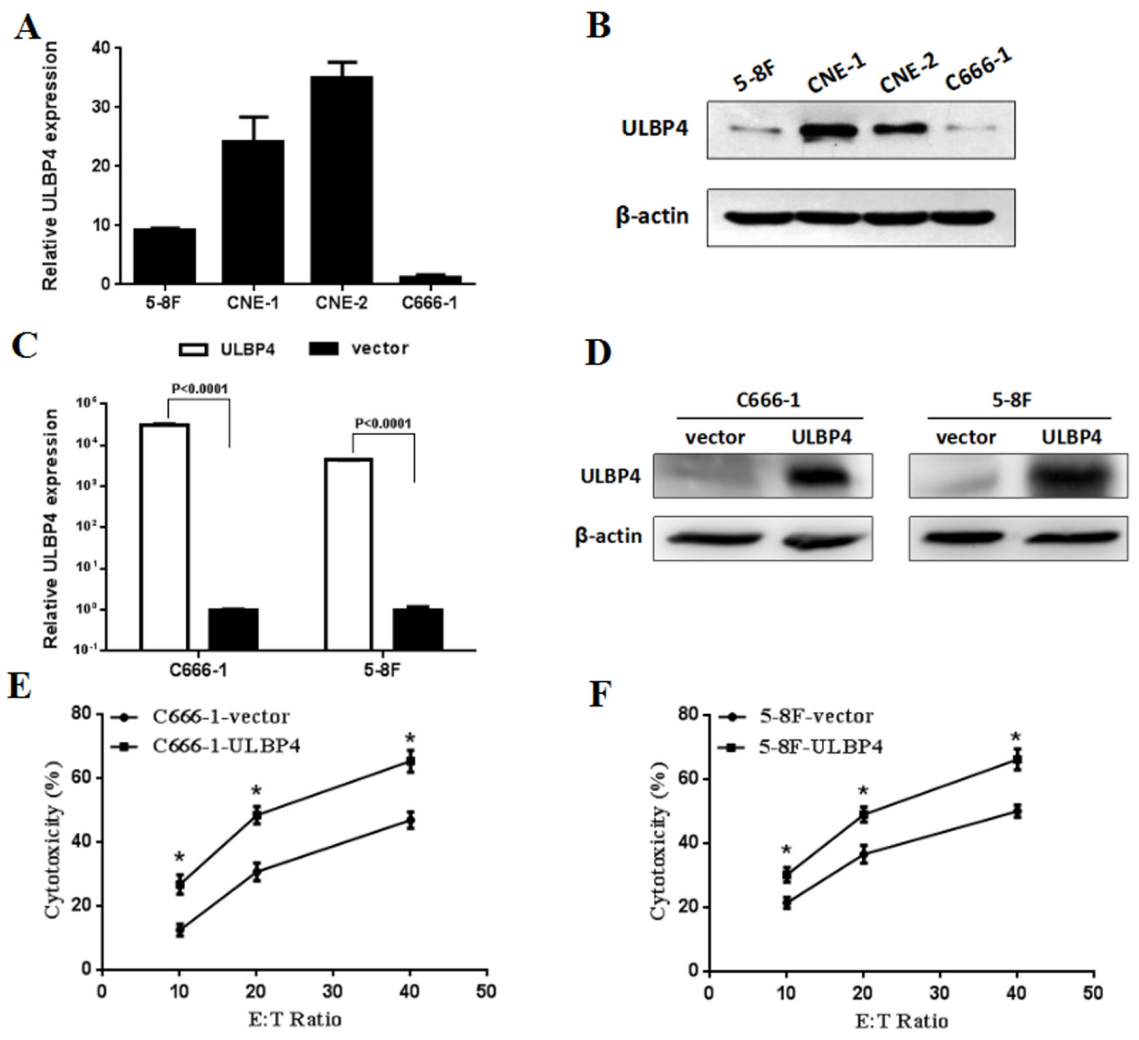
Impact of ULBP4 expression on the cytotoxic activity of NK cells *in vitro* **A**. qPCR analysis of ULBP4 expression levels among four NPC cell lines. **B**. Western blotting analysis of ULBP4 expression levels among four NPC cell lines. **C**. qPCR analysis of ULBP4 expression levels in C666-1 or 5-8F cells with lenti-ULBP4 compared with those with lenti-vector. **D**. Western blotting analysis of ULBP4 expression levels in C666-1 or 5-8F cells with lenti-ULBP4 compared with those with lenti-vector. **E**. LDH analysis of the cytotoxic activitity of NK cells against C666-1 with lenti-ULBP4 compared with that with lenti-vector at E/T ratios of 10:1, 20:1, and 40:1, respectively (*P < 0.05). **F**. LDH analysis of the cytotoxic activitity of NK cells against 5-8F with lenti-ULBP4 compared with that with lenti-vector at E/T ratios of 10:1, 20:1, and 40:1, respectively (*P < 0.05).

Due to low ULBP4 expression in NPC tissues, two NPC cell lines (C666-1, 5-8F) with relatively lower ULBP4 expression were determined to do further functional validation *in vitro* (Figure 4A and 4B). To investigate the effect of the ectopic ULBP4 expression on the cytotoxic activtity of NK cells, C666-1 and 5-8F cells were both infected with lentiviral particles carrying ULBP4 or its empty vector. Figure 4C and 4D showed a higher expression levels of ULPB4 after infection of ULBP4 in both C666-1 and 5-8F cells as compared to than in controls. In the LDH cytotoxicity analysis, the cytotoxic activitity of NK cells against NPC cell lines (C666-1 or 5-8F) with lenti-ULBP4 was significantly increased as compared to those with lenti-vector at the three E/T ratios (10:1, 20:1, and 40:1), respectively (all *P* <0.05, Figure 4E and 4F).

## DISCUSSION

The advent of IMRT combined with other new therapies has improved clinical outcomes in patients with NPC. Nevertheless, some cases tend to develop tumor progression and distant metastasis with the current treatment modalities. Though the TNM staging system for NPC is generally considered to be the gold standard to guide treatment and predict prognosis [21, 22], it may not be enough to provide optimal risk-stratification. Therefore, identification of additional prognostic biomarkers is required for guiding the individualized treatment. To our knowledge, this is the first study to explore the pattern of ULBP4 expression and its prognostic value in patients with NPC. In this study, ULBP4 was found to be significantly downregulated in primary NPC tissues when compared with normal NP tissues at both mRNA and protein levels. More importantly, low expression of ULBP4 was demonstrated to be of borderline significance in relation to death, disease failure and distant metastasis. Furthermore, low expression of ULBP4 in NPC cell lines attenuated the cytotoxic effect of NK cells *in vitro*, which was validated on LDH cytotoxicity analysis. These findings suggest that ULBP4 may prove to be a useful prognositc factor and a novel therapeutic target for NPC.

As a stress-inducible NKG2D ligand, ULBP4 is expressed in a variety of epithelial tumor cells, but is poorly expressed in normal non-tumor tissues [12–16, 18–20]. Conversely, our present study showed that ULBP4 was expressed at high levels in normal NP tissues rather than that in NPC tissues (Table 2, Figures 1 and 2). NPC is characterized by concomitant chronic inflammation, with the inflammatory cells releasing inflammatory factors such as IL-6 in NPC tissues, which promotes the development and progression of NPC [23, 24]. Under such conditions of stress, adjacent normal epithelial cells may also be stimulated by chronic inflammation to express high expression levels of ULBP4. Likewise, ULBP4 was found to be highly expressed on 8 normal fresh frozen NP tissues obtained from healthy individuals with chronic nasopharyngeal mucositis likely because of chronic inflammation stimulus. As high expression of ULBP4 on the surface of cells makes them more susceptible to be detected and killed by NK and T cells, it may prevent the transformation of adjacent normal epithelial cells into cancerous cells. In the present study, however, expression of ULBP4 in NPC cells was markedly decreased, which suggested that the down-regulation of ULBP4 may be one of the factors contributing to the immune evasion of NPC. Two possible mechanisms may explain the decreased surface expression of ULBP4 on NPC cells. Firstly, the ULBP4 expressed on the surface of tumor cells has been reported to be proteolytically cleaved by metalloproteases (MMPs) [25]. Such shedding could lead to downregulation of both the surface expression of ULBP4 on tumor cells and the expression of NKG2D receptor on effector cells, thus impairing NK cell cytotoxicity and T cell activation. Secondly, transforming growth factor-β (TGF-β), a tumor cytokine, has been reported to selectively suppress the transcription and protein expression of MICA, ULBP2 and ULBP4 on malignant glioma cells [26]. In addition, TGF-β may decrease the expression of NKG2D receptor on NK cells and T cells [27]. Secretion of TGF-β by NPC cells is well-documented and is known to promote the progression and metastasis of NPC [28, 29], herein ULBP4 expression may be also downregulated by TGF-β on NPC cells. However, definitive evidence of the specific underlying mechanisms involved in the negative regulation of ULBP4 expression in NPC tissues is still lacking.

**Figure 5:**
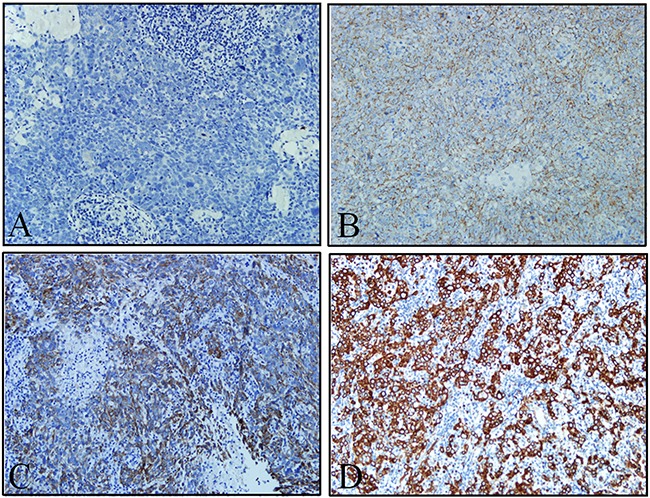
Criteria for ULBP4 staining intensity score **A**. Intenstiy score: 0. **B**. Intensity score: 1. **C**. Intensity score: 2. **D**. Intensity score: 3. The micrograghs were obtained and processed under the same conditions (original magnificaiton ×200).

Considering the heterogeneous expression of ULBP4 in primary NPC (Figure 5), we assessed the correlation between the deceased expression of ULBP4 and various clinicopathological characteristics. However, we found no significant association between the two (Table 1). These findings hinted that ULBP4 may serve as a novel biomarker independent of other clinical parameters. Moreover, the essential clinical parameters were comparable between the NPC patients with high and low expression of ULBP4, which allowed for a better comparison of the prognostic value between NPC patients with differential expression levels of ULBP4.

In spite of the heterogeneity in the surface expression of ULBP4 on NPC cells, univariate survival analysis showed that NPC patients with high expression of ULBP4 had better OS, PFS, and DMFS rates as compared to those associated with low expression of ULBP4 (Figure 3). These results indicated that the high expression of ULBP4 may serve to enhance host immune responses against NPC, thereby improving the prognosis in patients with NPC. This is in line with the theory that the high expression of NKG2D ligands may promote tumor immune surveillance by uniquely binding to the NKG2D receptor, and inducing the killing of cancer cells by NK cells and a subgroup of T cells [30]. Hence, expression levels of ULBP4 may serve as a criterion to identify patients with NPC at increased risk of tumor progression and distant metastasis. However, some reports found no evidence of the predictive effect of ULBP4 in colorectal cancer and early breast cancer [18, 20]. Furthermore, an inverse association of ULBP4 expression with the disease-free survival in cervical cancer and disease-specific survival in ovarian cancer has been reported [14, 19]. This could be explained by the overexpression of NKG2D ligands which may bring about an overstimulation effect, leading to insensitivity and anergy of NK and T cells, which in turn may contribute tumor immune evasion or immunoediting to some extent [30]. Taken together, these results hinted at diverse effects of ULBP4 in modulating anticancer immune responses depending on the type of cancer and the corresponding tissue microenvironment, which deserves to be investigated further.

On multivariate analysis, low ULBP4 expression was no longer associated with adverse OS, DFS, and DMFS rates in patients with NPC. Nevertheless, a borderline association with death (*P* = 0.060), disease failure (*P* = 0.053), and distant failure (*P* = 0.076) was observed (Table 4). This could be related to a favorable prognosis gained from IMRT and combined chemotherapy, which necessitates the enrollment of a larger number of patients with NPC to demonstrate the independent prognostic value of expression of ULBP4 in patients with NPC. ULBP4 may also prove to be an attractive target for developing an alternative therapy for NPC. Therapeutic strategies that reduce or block the proteolytic cleavage of soluble NKG2D ligands, for example, by inhibiting MMP activity to reduce the shedding of NKG2D ligands, may restore the expression of NKG2D receptor on immune effector cells and enhance their anticancer cytotoxicity [31]. In addition, reduction in tumor-induced immunosuppressive molecules such as TGF-β would increase the expression of NKG2D ligands on tumor cells, thereby improving the cytotoxic ability of NK cells and CD8+ T cells [32]. Therefore, future combined treatments using conventional cancer therapies and novel immunotherapies, towards enhancing the expression of ULBP4, may provide new therapeutic avenues in the prevention of tumor progression and distant metastasis in patients with NPC.

To further verify the impact of ULBP4 expression on the cytotoxicity activity of immune cells *in vitro*, our LDH cytotoxicity analysis demonstrated considerably increased the cytotoxic activity of NK cells against NPC cell lines (C666-1 or 5-8F) with lenti-ULBP4 as compared to those with lenti-vector at the three E/T ratios (10:1, 20:1, and 40:1), respectively (Figure 4). These results were consistent with previous studies that high ULBP4 expression could stimulate NK cell and NK-mediated cytotoxicity *in vitro* [11, 15, 16], which suggested that restoration of ULBP4 expression may be a novel therapeutic strategy for treatment of NPC. However, the impact of ULBP4 expression on cytoxicity of CD8+T or other T cells in NPC cell lines needs to be further investigated.

One of the limitations was that our current study was performed by retrospective analyses, and the number of our enrolled NPC patients seemed to be inadequate. In addition, concurrent chemoradiotherapay is still considered to be the standard therapeutic method for locally advanced NPC, even in the IMRT era. However, much heterogeneity with respect to the chemotherapy regimens existed in our study; only 58.1% patients received concurrent-based chemotherapy, which was due to participation of patients in the randomized clinical trials of certain chemotherapy regimens, or probably at the discretion of attending physicians in individual cases. Consequently, a prospective, randomized, and controlled clinical study with larger sample size is required to further resolve these issues.

To conclude, our study, perhaps for the first time, demonstrated decreased expression levels of ULBP4 in NPC tissues as compared to that in normal NP epithelial tissues. In addition, low ULBP4 expression correlated with poor OS, DFS, DMFS, and may prove to be a novel prognostic factor. Low ULBP4 expression in NPC cell lines could attenuate the cytotoxicity of NK cells *in vitro*. Restoration of ULBP4 expression may be a novel therapeutic target for NPC, which however warrants further investigation.

## MATERIALS AND METHODS

### Patients and specimens

The study was conducted on a series of 111 formalin-fixed, paraffin-embedded samples obtained from patients with newly diagnosed and histologically proven cases of NPC, but without distant metastasis, in Fujian Provincial Cancer Hospital, between January and December 2010. All NPC specimens were obtained prior to initiation of chemotherapy or radiotherapy. All patients underwent MRI scan of the nasopharynx and neck; the pretreatment evaluation was based on the institutional protocol [5]. All patients had a satisfactory Karnofsky performance status score (> 70). The study exclusion criteria included a prior or synchronous malignancy. Disease staging was performed according to the 7^th^ edition of the International Union Against Cancer/American Joint Committee on Cancer (UICC/AJCC) staging system for NPC [21, 22]. An additional fresh frozen samples (15 primary NPC tissues from NPC patients and 8 nasopharynx (NP) tissues from healthy individuals with chronic nasopharyngeal mucositis) archived in 2012 were also obtained from our hospital. All procedures were conducted after obtaining due approval of the Institutional Review Board at the Fujian Provincial Cancer Hospital, China (reference number 2015-010-02).

### Cell line and cell culture

The human cell lines CNE-1, CNE-2, 5-8F, and C666.1 were all maintained in RPMI 1640 medium (Gibco, Grand Island, NY, USA) supplemented with 10% fetal bovine serum (Gibco, Australia). Peripheral blood mononuclear cells (PBMCs) were isolated from the whole blood of healthy individuals by Ficoll density gradient centrifugation, then NK cells were cultured in lymphocyte serum-free medium KBM 581 (Corning, NY, USA) and were amplified by a combination of IL-2 (500IU/mL), IL-12 (2 ug/mL), IL-15 (2 ug/mL), and IL-18 (10 ug/mL) every other day for a total of 14-17 days. Ultimately, highly pure NK cells were isolated by CD56 MicroBeads (Miltenyi Biotec Inc, Auburn, CA, USA) with VarioMACS system. The purity CD3-/CD56+ NK cells was assessed to be 90%-95% on flow cytemerty.

### RNA extraction and quantitative real-time PCR (qPCR)

Total RNA from 15 NPC, 8 normal NP tissue specimens and NPC cell lines was extracted by TRIzol reagent (Invitrogen), and reversely transcribed with the PrimeScript RT reagent kit (TaKaRa, Japan). qPCR was performed on a LightCycler Roche 480 using DyNAmo Flash SYBR-Green qPCR kit (Thermo Fisher Scientific, USA) according to the manufacturer's standard manual. Primer sequences used for ULBP4 detection were as follows: forward primer: 5′-CGCCTTCTTTTGTTTCTGCTG-3′, reverse primer: 5′-CCTGAGGTCTCGCCCCACT-3′. β-actin was used as the internal control, and its primer sequences were set as follows: forward: 5′-TGACGTGGACATCCGCAAAG-3′, reverse: 5′-CTGGAAGGTGGACAGCGAGG-3′. Relative quantification of mRNA expression was performed by the comparative 2^−ΔΔct^ method to calculate the difference between amplifications of ULBP4 and β-actin RNA. The experiments were performed with three technical replicates, and the mean fold changes and standard deviation were also calculated.

### Immunohistochemical study

Expression of ULBP4 in NPC tissue samples was assessed on immunohistochemistry (IHC) examination. Paraffin-embedded NPC specimens were sliced into 4-μm thick sections, deparaffinised in xylene and rehydrated through a graded ethanol series. Next, the slides were boiled in a pressure cooker until gas discharging for 2.5 min in citrate buffer (Maixin-bioMVS-0101) (pH 6.0). Endogenous peroxidase was blocked by treatment with 3% hydrogen peroxide (H_2_O_2_) for 10 min. Subsequently, sections were incubated with mouse anti-human ULBP4 monoclonal antibody (1:1000, R&D Systems, Inc., Minneapolis, MN, USA) for 1h at 37°C; positive and negative controls were also used. After washing with phosphate-buffered saline (PBS) 3 times, tissues were incubated with horseradish peroxidase-conjugated anti-mouse Ig polymer as a second antibody (Elivision kit, Maixin-Bio, Fuzhou, People's Republic of China) for 30 min at 37°C. Finally, sections were visualized using diaminobenzidine (DAB) staining kit, counter-stained with hematoxylin, and observed under the microscope.

### Evaluation of IHC staining

To determine the expression of ULBP4, all slides were stained under the same conditions using a standard protocol. ULBP4 is primarily expressed on membrane of epithelial cells, but seldom shows a cytoplasmic pattern. The expression levels of ULBP4 were assessed by two scores, i.e., the staining intensity and the percentage of the stained epithelial cells. Considering that the staining of tumors was relatively heterogeneous, the immunoreactive scores of ULPB4 expression were determined as previously described [14]. Briefly, the staining intensity was scored as negative (0), weak (1), moderate (2), or strong staining (3) (as shown in Figure 5). The percentage of positive staining was scored as 0% (0), < 25% (1), 25%-50% (2), 50.1%-75% (3), or > 75% (4). The overall score was calculated by multiplying intensity score and percentage score, which ranged from 0 to 12. All slides were evaluated by two independent pathologists in a double-blinded manner. Any slides with conflicting scores were resolved by consensus.

### Western blotting

Western blotting analyses were conducted with the standard protocols. In brief, human NPC cell lines were lysed in modified lysis buffer (Roche) for 15 minutes on ice. The BCA Protein Assay kit (Pierce, USA) was used to determine cellular protein concentration. Then total protein from cells was extracted by loading buffer (0.2% bromophenol blue, 20% glycerol, 125 mM Tris-HCl, 640 mM βME, 4% SDS), and boiled for 10 minutes. After that, total proteins were separated on 10% SDS-PAGE gels and electro-transferred to membranes (Millipore). Then the membranes were probed with rabbit polyclonal anti-ULBP4 antibody (1:500; Abcam, Chicago, IL, USA) at 4°C overnight, and then with anti-rabbit IgG secondary antibody (1:5000; Sigma, St. Louis, MO, USA). β-actin was used as protein loading controls.

### Plasmid construction and lentivirus transduction

To obtain NPC cell lines to overexpress ULBP4, the sequence of ULBP4 was cloned into the pLVTHM lentiviral vector, and the recombinant plasmid was renamed as lv-ULBP4. The lentiviral viruses overexpressing ULBP4 (lenti-ULBP4) and the nagative control lenti-vector (lenti-vector) were purchased from Life-Int (Xiamen, China). These were used to infect C666-1 and 5-8F NPC cell lines according to the manufacturer's recommended protocol. All cell qPCR, Western blotting, and functional studies were performed at least 72 hours after lentiviral infection.

### Cytotoxicity assay

Lactate dehydrogenase (LDH) assay was performed to reflect the cytotoxic activity of effector cells on target cells using a nonradioactive cytotoxicity assay kit (Promega, Madison, WI, USA). Generally, the target cells (C666-1, 5-8F), with lenti-vector or lenti-ULBP4, were set in triplicate in 96-well culture plates and incubated with the effector cells (NK cells) with an effector to target (E/T) ratio of 10:1, 20:1 or 40:1. Maximal release of LDH was conducted by complete lysis of target cells. Target cells without effector cells were considered as negative controls (spontaneous release). The cytotoxic effect was measured as follows: percentage cytotoxicity (%) = [(experimental release - spontaneous release of effector cells - spontaneous release of target cells) / (maximal release of target cells - spontaneous release of target cells)] x100.

### Treatment

All 111 patients were initially treated with definitive IMRT. The details of the IMRT have been previously described [5]. Of the 108 NPC patients with Stages II-IVB disease, 98 (90.7%) received platinum-based chemotherapy. The sequence administered was induction in 10 (10.2%), concurrent in 11 (11.2%), concurrent-adjuvant in 5 (5.1%), induction-concurrent in 19 (19.4%), induction-adjuvant in 31 (31.6%), and induction-concurrent-adjuvant in 22 (22.4%) patients. Whenever possible, salvage treatment with transcavitary brachytherapy, surgery, and chemotherapy were administered in case of recurrence or disease persistence.

### Follow-up and statistical analyses

The duration of follow up was calculated from the date of diagnosis to death or to the last follow up examination for those still alive. Generally, after the completion of therapy, patients were followed up every 3 months during the first 2 years, every 6 months from year 2 to year 5, and annually thereafter. The median follow-up time for the whole group was 66 months (range, 7-74 months). Overall survival (OS), progression-free survival (PFS), local relapse-free survival (LRFS), and distant metastasis-free survival (DMFS) was calculated from the initial diagnosis to death, disease failure, local failure, or distant failure. For patients who were still alive at the last follow up, the duration of survival was censored.

All statistical analyses were performed using the version 17.0 of Statistical Package for the Social Science software (SPSS, Inc, Chicago, IL, USA). *t* test was employed to compare mRNA expressions of ULBP4 in the tumor and non-tumor tissue specimens, and to compare the cytotoxic activity of NK cells against NPC cell lines with lenti-ULBP4 and lenti-vector. Chi-squared test was used to compare the ULBP4 expression in cancer cells with that in adjacent normal nasopharyngeal epithelia, and to evaluate the association between the expression of ULBP4 and clinicopathological characteristics. Survival rates were calculated by the Kaplan-Meier method and the statistical significance of differences were assessed by log-rank test. Multivariate analyses using the Cox proportional hazards model were conducted to explore the independent prognostic factors for the whole cohort. In all cases, a two-tailed *P* value of <0.05 was considered statistically significant.
